# Insight into the Regulation of NDRG1 Expression

**DOI:** 10.3390/ijms26083582

**Published:** 2025-04-10

**Authors:** Concetta Saponaro, Nicola Gammaldi, Viviana Cavallo, Maria Antonieta Ramírez-Morales, Francesco Alfredo Zito, Margherita Sonnessa, Francesco Vari, Ilaria Serra, Simona De Summa, Anna Maria Giudetti, Marco Trerotola, Daniele Vergara

**Affiliations:** 1 Pathology Department, IRCCS Istituto Tumori “Giovanni Paolo II”, 70124 Bari, Italy; c.saponaro@oncologico.bari.it (C.S.); a.zito@oncologico.bari.it (F.A.Z.); m.sonnessa@oncologico.bari.it (M.S.); 2 Department of Biological and Environmental Sciences and Technologies (DiSTeBA), University of Salento, 73100 Lecce, Italy; nicola.gammaldi@unisalento.it (N.G.); viviana.cavallo@unisalento.it (V.C.); mariaantonieta.ramirezmorales@unisalento.it (M.A.R.-M.); francesco.vari@uniroma1.it (F.V.); ilaria.serra@uniroma1.it (I.S.); anna.giudetti@unisalento.it (A.M.G.); 3 Department of Physiology and Pharmacology “V. Erspamer”, Sapienza University of Rome, P.le Aldo Moro 5, 00185 Rome, Italy; 4 Molecular Diagnostics and Pharmacogenetics Unit, IRCCS Istituto Tumori, “Giovanni Paolo II”, 70124 Bari, Italy; s.desumma@oncologico.bari.it; 5 Laboratory of Cancer Pathology, Center for Advanced Studies and Technology (CAST), “G. d’Annunzio” University of Chieti-Pescara, 66013 Chieti, Italy; marco.trerotola@unich.it; 6 Department of Medical, Oral and Biotechnological Sciences, “G. d’Annunzio” University of Chieti-Pescara, 66013 Chieti, Italy

**Keywords:** NDRG1, cellular stress, protein regulation, cellular signalling, phosphorylation

## Abstract

The N-Myc Downstream Regulated Gene 1 (NDRG1) protein, a member of a family of four, has emerged as a key regulator of various physiological and pathological processes. Extensive knowledge has been gained on the modulation of NDRG1 expression during endoplasmic reticulum stress, autophagy, and hypoxia. Moreover, new functions have emerged in recent years. Notably, NDRG1 regulates cell differentiation, metabolism, autophagy and vesicular transport. This has raised interest in the molecular mechanisms that control the cellular levels and activity of NDRG1. A series of studies have shown that NDRG1 can be finely regulated at the transcriptional, post-transcriptional, and translational levels. In addition, processes that mediate protein degradation and clearance also play key roles. Furthermore, three different NDRG1 proteoforms with distinct functions have been identified. An important question is the extent to which these proteoforms contribute to the regulation of cellular functions. Given the growing clinical interest in NDRG1, this review provides an overview of the regulatory mechanisms that control NDRG1 abundance, helping to deepen our understanding of the complex mechanisms underlying protein regulation.

## 1. Introduction

N-myc downstream regulated 1 (NDRG1) (formerly known as Drg1, Cap43, Rit42, RTP, and PROXY-1) is a 43 kDa protein (UniProt: Q92597) that belongs to a family of α/β hydrolase proteins comprising four members: NDRG1, NDRG2, NDRG3, and NDRG4. The *NDRG1* gene comprises 17 exons on chromosome 8 (8q24.22). The other family members, NDRG2, NDRG3, and NDRG4 are located at chromosome 14q11.1–11.2, 20q12–11.2,3, and 16q21–22.1, respectively [1]. These four isoforms have structural homologies and differences, sharing 53% to 62% structural identity with human NDRG1 [1]. For example, all NDRG isoforms express an α/β hydrolase domain, although none possess a hydrolytic catalytic function. Specifically, NDRG1 comprises an N-terminal region (residues 1–30), followed by a central α/β hydrolase domain, including a cap region (residues 31–310). Analysis of the primary structure revealed a unique characteristic of NDRG1, consisting of three 10-amino acid (GTRSRSHTSE) tandem repeats (residues 339–368) in the C-terminal region, which are absent in NDRG2, NDRG3, and NDRG4 [1]. Each repeat contains a metal-binding site for nickel (II), copper (II), zinc, manganese, and cobalt [2,3]. It has been proposed that metal binding may be crucial in regulating NDRG1 expression and potentially influencing detoxification processes. It is unclear whether metal binding provides a pathway for protein stabilisation or a stimulus for the activation of signalling pathways leading to the modulation of NDRG1 expression. This hypothesis is supported by several studies. For instance, nickel (II) appears to induce NDRG1 expression by inducing hypoxia and elevating free intracellular Ca^2+^ levels [4,5].

Furthermore, the C-terminal domain can act as a substrate for phosphorylation by various kinases, including protein kinase C (PKC), casein kinase II (CK2), and serum and glucocorticoid-induced kinase 1 (SGK1). Phosphorylation of the C-terminal region regulates NDRG1 localisation and function [6].

In addition, the C-terminal region is responsible for binding to various phosphatidylinositol phosphates (PIPs), including PI4P, PI (3,5)P2, PI (4,5)P2, and PI (3,4,5)P3 [7]. Phosphoinositides are signalling precursors and regulators of several cellular processes, such as membrane trafficking and polarity. The binding of NDRG1 to this class of lipids is important for regulating their cellular functions.

**Figure 1:**
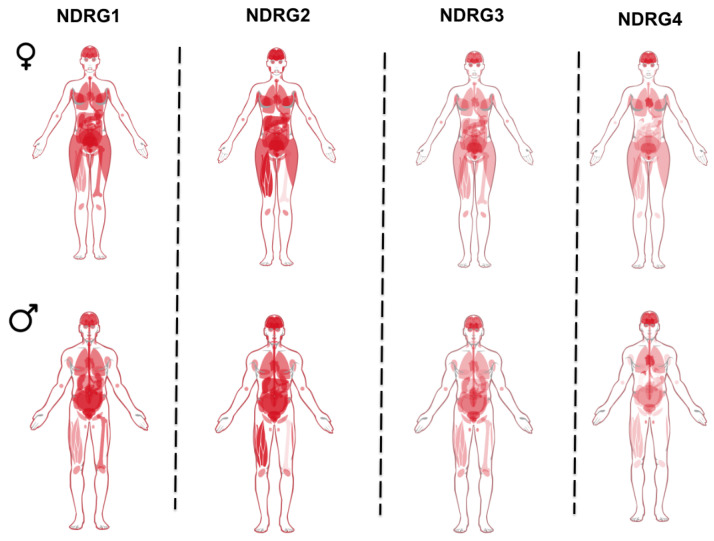
The protein expression profiles of NDRG family members in human tissues. Data were obtained from the Human Protein Atlas (https://www.proteinatlas.org/, accessed on 13 January 2025).

Regarding the expression of NDRG members, NDRG1 is ubiquitously expressed, while the other isoforms appear to be specific to certain tissues (Figure 1). For instance, the expression of NDRG4 was quite specific to the brain and heart [8]. In the brain, the expression levels of NDRG1, NDRG2, and NDRG3 were not affected by the lack of NDRG4, suggesting that there are no compensatory mechanisms of gene expression for the members of this family [9]. Recently, the expression of NDRG4 was also observed in the enteric nervous system of both mice and humans [10]. As shown in Figure 1, NDRG1 expression appears to vary between men and women, indicating that NDRG1 may respond to tissue- and sex-specific regulatory mechanisms in the testis. For instance, data extracted from the Sex Associated Gene Database (SAGD) demonstrate significant differences in NDRG1 expression in various tissues between men and women. These include the liver (−1.1885 log2 (M/F ratio)), gonads (−2.1594 log2 (M/F ratio)), long bones (3.9405 log2 (M/F ratio)), and myoblasts (2.834 log2 (M/F ratio)) (https://doi.org/10.1093/nar/gky1040, accessed on 13 January 2025). A sex-discriminating signature is also present in pathophysiological contexts. Data gathered from the SexAnnoDB website confirmed that NDRG1 expression is associated with sex. For example, in the case of breast cancer, expression is higher in female tumour tissues than in male tissues (Log2foldchange −1.14 [11]. Moreover, the influence of sex differences on NDRG1 expression is supported by studies that highlighted the functional relationship between NDRG1 expression and hormonal status. For instance, research has indicated that NDRG1 can be regulated by androgens, suggesting a potential link between sex and specific biological processes [12]. Furthermore, studies on breast cancer (BC) have shown an inverse correlation between NDRG1 expression and estrogen receptor-alpha (ER-α) expression [13]. This finding implies that hormonal influences may play a role in modulating NDRG1 levels. Three different isoforms of NDRG1 have been identified and produced via alternative splicing. Isoform Q92597-1 represents the canonical sequence with a length of 394 amino acids, isoform Q92597-2 lacks a 1–66 amino acid region, and isoform Q92597-3 lacks a 1–81 amino acid region, both compared to the canonical sequence (UniProt data: https://www.uniprot.org/uniprotkb/Q92597/entry, accessed on 13 January 2025). NDRG isoforms are present at distinct subcellular locations. The full-length NDRG1 isoform localises extensively in the nucleus compared to the truncated isoforms, indicating that the full-length isoform may translocate to the nucleus from the cytosol. In contrast, truncated isoforms may be unable to translocate [6].

Several studies also support NDRG1 localisation in vesicles isolated from different cell/tissue types (Vesiclepedia data: http://microvesicles.org/gene_summary?gene_id=10397, accessed on 13 January 2025). Further studies are required to establish the functional role of this specific localisation. This protein has also been detected in plasma at a concentration of 110 ng/L (https://www.proteinatlas.org/ENSG00000104419-NDRG1/blood, accessed on 13 January 2025), as determined by mass spectrometry. Plasma concentrations of NDRG1 in the blood of patients with different disease types, as measured by proximity extension assay (PEA), showed that expression levels might vary depending on the specific pathological condition (https://www.proteinatlas.org/ENSG00000104419-NDRG1/blood, accessed on 13 January 2025). In the work of Wang and collaborators, NDRG1 expression levels were determined in the serum of control and lung cancer patients using ELISA. Serum levels in lung cancer patients were significantly higher (358.56 ± 233.82 ng/mL) than those in healthy controls (28.83 ± 10.51 ng/mL) [14]. Moreover, data extracted from the Atlas Proteome Project demonstrated that NDRG1 is linked to the incidence of disease risk and is associated with specific health-related traits. For instance, the top associations were mainly found in acute renal failure (*p* = 5.4988 × 10^−9^) and chronic kidney disease (*p* = 2.8494 × 10^−8^). In protein-trait associations, NDRG1 was associated with blood and urine assays, especially cystatin C, NMR metabolomics, especially lactate, and physical measures, including the body mass index [15].

## 2. Regulation of NDRG1 Expression

NDRG1 was initially identified as a gene target repressed by the oncoprotein N-Myc [16]. Early works have associated NDRG1 expression with growth arrest or cell stress. In the U937 cell line, NDRG1 expression was induced by retinoids, vitamin D3, and phorbol ester. Agents that can modulate the proliferative state of cells by inducing differentiation [17]. NDRG1 mRNA expression was induced alongside genes involved in endoplasmic reticulum stress, such as GRP78/BiP, in HUVEC cells [18]. Additionally, overexpression of NDRG1 has been observed in cells cultured under hypoxic conditions [19]. These studies suggest a potential role for NDRG1 expression in regulating cellular stress response pathways.

Different molecular mechanisms are involved in modulating NDRG1 levels in response to cellular perturbations, including (i) epigenetic regulation through chromatin methylation, (ii) transcriptional control by specific transcription factors, (iii) translational regulation via the formation of specialised translation machinery under stress conditions or through microRNAs (miRNAs), and (iv) post-translational regulation by reversible phosphorylation/dephosphorylation or protein degradation, mediated by the ubiquitin-proteasome system (UPS) and lysosomal proteolysis pathway.

**Epigenetic regulation.** Epigenetic modifications are crucial for NDRG1 expression. In breast cancer (BC), loss of *NDRG1* mRNA expression is directly correlated with aberrant methylation and tumorigenesis [20]. Mircetic and collaborators demonstrated that the histone lysine demethylase-1A (KDM1A) regulates the expression of *NDRG1* in a gastric cancer (GC) organoid model through histone demethylation. Specifically, KDM1A forms a regulatory loop with the REST/RCOR2 complex, which recruits KDM1A to the *NDRG1* promoter. Genetic and pharmacological inhibition of KDM1A leads to a decrease in the growth of GC organoids through NDRG1/Wnt signalling inhibition [21].

**Transcriptional regulation**. Several transcription factors are known to induce upregulation of NDRG1, including the transcriptional coactivator YAP1 (YAP), tumour suppressor p53 (p53), hypoxia-inducible factor 1 alpha (HIF1ɑ), zonula occludens 1 (ZO-1)-associated nucleic acid-binding (ZONAB) protein, eukaryotic Initiation Factor 3a (eIF3a), and activating transcription factor 4 (ATF4) [22,23,24,25,26,27]. On the contrary, NDRG1 is negatively regulated by estrogen receptor-alpha (ER-ɑ). Treatment with 17beta-estradiol decreased the expression of NDRG1 dose-dependently in ER-ɑ-positive cell lines [13]. NDRG1 regulation by YAP has been extensively studied. YAP can regulate NDRG1 expression by directly binding to the NDRG1 gene promoter or indirectly by regulating other proteins. A study on MDA-MB-231 and BT549 BC cells demonstrated a functional correlation between YAP and NDRG1 expression. After its activation and nuclear translocation, YAP binds to the 0–191 region of the *NDRG1* promoter, regulating its transcription. In addition, YAP knockdown decreases NDRG1 expression [27].

YAP can also indirectly regulate NDRG1 expression by controlling the activity of kinases, such as SGK1. mTORC2 activates YAP through phosphorylation at Ser436, leading to increased expression of YAP target genes, including SGK1 [28,29], which is responsible for NDRG1 phosphorylation [30]. SGK1 activation is functionally important for supporting cell growth under stressful conditions, including endoplasmic reticulum (ER) stress and mitochondrial dysfunction. In BC cells, the ER stress inducer thapsigargin, but not tunicamycin, increases *SGK1* mRNA expression, suggesting that specific ER stimuli are required to upregulate SGK1 transcription. SGK1 expression is specifically regulated by Ca^2+^ influx from the extracellular milieu following Sarco-Endoplasmic Reticulum Calcium ATPase (SERCA) inhibition by thapsigargin and requires calmodulin and CaMKII as key downstream effectors of intracellular Ca^2+^ signal transduction. A relative increase in the percentage of cells undergoing necrosis was observed in cells expressing short hairpin RNA targeted to the *SGK1* gene [31]. Although SGK1 is an upstream regulator of NDRG1, the two proteins follow different response mechanisms after treatment with ER-inducing agents. NDRG1 overexpression is induced in response to both tunicamycin and thapsigargin treatment [32].

Stimuli from the microenvironment may converge on the YAP/NDRG1 regulatory axis. Components of the extracellular matrix can bind to integrins and transmit mechanical signals, which in turn promote the upregulation of YAP and the expression of stemness-related genes, including NDRG1 [33].

**Figure 2:**
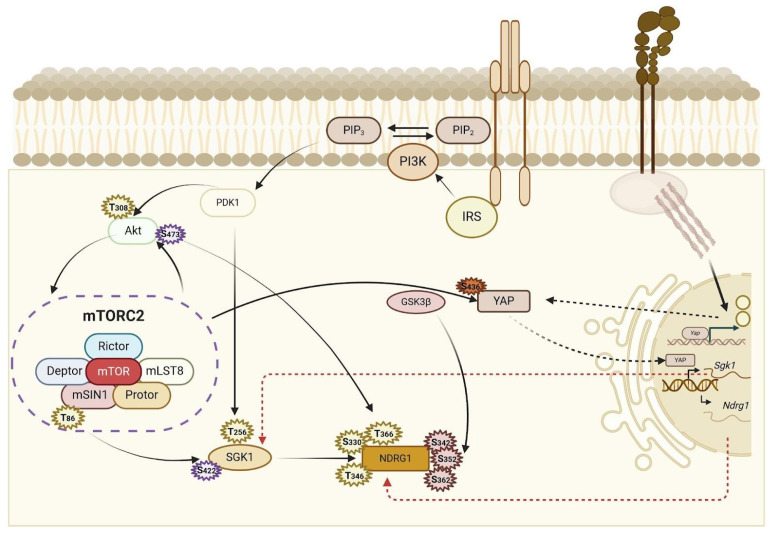
A representative image of the cellular mechanisms controlling the phosphorylation and expression of the NDRG1 gene. NDRG1 phosphorylation is regulated by SGK1, which in turn is phosphorylated by mTORC2. Furthermore, mTORC2-mediated phosphorylation of YAP can lead to the nuclear translocation of YAP and transcriptional regulation of *SGK1* and *NDRG1*. YAP-mediated regulation of NDRG1 may also involve a mechanotransduction signal involving membrane integrins and the extracellular matrix. Abbreviations: DEP domain-containing mTOR-interacting protein (DEPTOR), insulin receptor substrate (IRS), mammalian stress-activated protein kinase [SAPK]-interacting protein (mSIN1), N-Myc downregulated gene 1 (NDRG1), mammalian lethal with SEC13 protein 8 (mLST8), phosphatidylinositol 4,5-bisphosphate (PIP2), phosphatidylinositol (3,4,5)-trisphosphate (PIP3), 3-phosphoinositide-dependent protein kinase-1 (PDK1), phosphoinositide 3-kinase (PI3K), protein kinase B (AKT), mechanistic target of rapamycin complex 2 (mTORC2), protein observed with Rictor-1 (PROTOR), RPTOR independent companion of MTOR complex 2 (RICTOR), serum- and glucocorticoid-induced protein kinase 1 (SGK1), transcriptional coactivator YAP (YAP). Created in BioRender. Azzariti, A. (2025) https://BioRender.com/j39e614 (accessed on 13 January 2025).

Based on these observations, it is plausible to hypothesise that increased SGK1 expression through mTORC2 activation of YAP or after stress conditions represents a feed-forward loop to sustain NDRG1 phosphorylation (Figure 2).

**p53.** NDRG1 expression is induced in a p53-dependent manner [22]. DNA-damaging agents or factors that affect cell proliferation activate p53, which binds to the promoter region of *NDRG1* at 406 bp upstream of the transcriptional initiation site of NDRG1, thereby regulating its mRNA expression [34]. These results may be particularly relevant in tumours where a mechanistic link between p53, NDRG1, and centrosome homeostasis has been described [35]. In vitro data demonstrated that the loss of p53 in cells undergoing low proliferation fails to upregulate NDRG1, dysregulating normal centrosome homeostasis. In vivo, a potential inverse relationship between homozygous loss of *TP53* and *NDRG1* overexpression has been observed across various cancer types [35]. A mechanistic role for NDRG1 has also been proposed. NDRG1 was found to interact with γ-tubulin, directly regulating normal versus abnormal centrosome numbers [35].

NDRG1 is necessary for the proper p53 mitotic spindle checkpoint, as NDRG1 inhibits polyploidy and increases the population of cells in mitotic arrest in several p53-deficient tumour cell lines. Inhibition of this gene using a short interfering RNA (siRNA) in normal human mammary epithelial cells increases the polyploidy state after nocodazole treatment [36].

**HIF-1.** HIF-1 is an oxygen-regulated transcriptional activator that drives the expression of a hypoxic gene set in cells exposed to a reduced-oxygen environment. HIF-1 activation during hypoxia regulates cellular pathways that affect gene expression, metabolism, and cell survival, thus promoting tumorigenesis and tumour growth. In vitro and ex vivo studies have demonstrated that *NDRG1* gene expression is regulated by hypoxia and hypoxia-mimicking transition metals (nickel and cobalt) [23,37]. *NDRG1* gene induction primarily depends on HIF-1, as neither RNA nor NDRG1 protein was induced in HIF-1α^−/−^ cells upon short-term exposure to hypoxia [23]. A reporter assay showed that HIF-1α could activate gene expression by binding to the *NDRG1* promoter [38]. However, other regulatory mechanisms involved in the upregulation of NDRG1 under hypoxic conditions cannot be excluded. Some of these mechanisms involve multiple transcription factors activated by biological processes, endoplasmic reticulum stress, and calcium signalling pathways that are modulated in response to hypoxia. Importantly, as described later, a further level of regulation of hypoxic genes is achieved through mRNA stabilisation.

In addition to HIF-1α, other transcription factors involved in hypoxia signalling can regulate NDRG1 expression. A signal pathway sensitive to hypoxia is the (ER) stress through the activation of the unfolded protein response (UPR) pathway. This pathway regulates the activation of transcription factors that control the expression of NDRG1, including XBP1 and ATF4 [39]. Furthermore, a study on RAW264.7 cells grown under hypoxic conditions demonstrated that the transcription factor Egr-1 is required for NDRG1 induction. Egr-1 functions as a positive regulator of NDRG1 expression by binding to an Egr-1/Sp1 binding motif in the *NDRG1* proximal promoter [40].

An HIF-independent mechanism has also been proposed for the upregulation of NDRG1. Under hypoxic conditions, the elevation of intracellular calcium triggers the activation of activator protein 1 (AP-1), which binds to the element in the 5′-end of the *NDRG-1/Cap43* gene. This leads to *NDRG-1* mRNA expression in HIF-1 deficient cells [41].

Overall, NDRG1 expression is regulated by HIF. However, ectopic overexpression of NDRG1 does not trigger a gene expression profile resembling the hypoxic response, showing that NDRG1 is not a regulator of hypoxia-induced gene signature [42]. Moreover, the response to hypoxia is specific to NDRG1 because NDRG2–4 are not differentially regulated under this condition [43].

Considering that NDRG1 is activated by hypoxia but that the gene alone is not capable of inducing a hypoxic signature, several authors have questioned the functional role of NDRG1 in the hypoxic context. An elegant work conducted on Zebrafish assigns to NDRG1 a specific role in this scenario. Under hypoxic conditions, NDRG1a, the ortholog of mammalian NDRG1, promotes organismal survival and protects the kidney from hypoxic injury following exposure to prolonged anoxia. Activated NDRG1a accelerates the endocytosis and degradation of the energy-demanding sodium-potassium ATPase (NKA) pump in the kidney and monocytes to preserve ATP [44].

**ZONAB**. In colorectal cancer (CRC), NDRG1 downregulation was found to be essential for driving Claudin-2 (CLDN2)-mediated cell proliferation and migration/invasion in vitro and in vivo. CLDN2 suppresses NDRG1 expression in CRC, while CLDN2 depletion induces NDRG1 expression, leading to reduced colony-forming ability and proliferation. This process is regulated by CLDN2/ZO1/ZONAB complex dissociation and the migration of ZONAB into the nucleus. When ZONAB shuttles into the nucleus, it binds to the promoter region of *NDRG1,* regulating its transcription [26].

**Post-translational regulation**. NDRG1 is phosphorylated at several sites, including Thr328, Ser330, and Thr346/Thr356/Thr366 by SGK1, which in turn is regulated by mTORC2. NDRG1 phosphorylation is widely used as a readout for SGK1 and mTORC2 activity [30].

This protein is a member of the protein kinase A, G, and C (AGC) family. The activation of SGK1 requires phosphorylation at Ser422 and Thr256 by mTORC2 and PDK1, respectively, upon stimulation of phosphoinositide 3-kinase (PI3K) signalling and insulin (Figure 2) [30]. In the kidney, Protor-1, a subunit of mTORC2, plays a role in enabling mTORC2 to efficiently activate SGK1. *Protor-1* knockout mice displayed markedly reduced hydrophobic motif phosphorylation of both SGK1 and NDRG1 [45].

Knockdown of SGK1 in HeLa cells greatly reduced the phosphorylation of NDRG1 at the sites targeted by SGK1. Moreover, phosphorylation by SGK1 primes NDRG1 for phosphorylation by glycogen synthase kinase 3B (GSK3B) at Ser342, Ser352, and Ser362 [46]. In head and neck cancer cell lines, NDRG1 phosphorylation by GSK3β is critical for its nuclear import [47].

The mTORC2/SGK1/NDRG1 pathway responds to various cellular stress conditions and is associated with signalling pathways. For instance, Akt and AMPK co-regulate mTORC2, enhancing its activity and NDRG1 phosphorylation. One mechanism involves the direct phosphorylation of SIN1, a subunit of mTORC2, at Thr86 by Akt in response to a wide range of external stimuli. Akt then requires a second phosphorylation event at Ser473 by mTORC2 for its full activation [48]. Moreover, under conditions of energetic stress, including serum deprivation and glucose withdrawal, AMPK directly phosphorylates mTORC2 at Ser1261 to promote cell survival [49]. Functionally, this leads to increased cell survival by suppressing the stress-induced apoptosis. Additionally, other stress conditions, such as loss of integrin signalling, can activate the SGK1/NDRG1 signalling axis to promote cell survival by increasing glucose entry via the GLUT1 receptor, thereby increasing the cellular metabolic substrates [50].

Nutrients can also activate the mTORC2. Ammonia-induced mTORC2 activation and NDRG1 phosphorylation at Thr346 depend on YES1, a member of the non-receptor tyrosine kinase SRC family of kinases, and focal adhesion kinase (FAK) [51].

mTORC2/SGK1 is an essential upstream regulator of NDRG1. However, activation can also occur through other signalling pathways. In BC cells, an enhanced expression of phosphorylated NDRG1 at Thr346 was observed after Tumour Growth Factor-β1 (TGFβ1) treatment. TGFβ1 signals through the activation of GSK3β (phosphorylated at Tyr216) [52]. BC cells displaying high Akt activity and low SGK1 expression still maintain a high level of phosphorylated NDRG1 at Thr346. In these models, Akt inhibitors suppress NDRG1 phosphorylation, suggesting a direct role for Akt in supporting NDRG1 phosphorylation [53].

Phosphorylation of NDRG1 may impact its localisation, interaction with other cellular proteins, and function. For instance, phosphorylated NDRG1 was found to co-localise with γ-tubulin on centromeres or to recruit cell division cycle 42 (CDC42) to mitochondrial–endoplasmic reticulum contacts [54,55].

**Translational efficiency.** An increased half-life of the NDRG1 protein relative to its mRNA has been described. Indeed, after reoxygenation, mRNA levels returned to near-control values within 2 h while protein levels remained high 72 h after re-exposure to 20% oxygen [19]. This suggests that the mechanisms controlling protein synthesis may represent important layers of NDRG1 expression regulation. Translation efficiency can be controlled at any phase by various mechanisms. During the initiation phase, two distinct cap-dependent protein synthesis machineries regulate this process: normoxic eIF4F and hypoxic eIF4F^H^. These two protein complexes can recruit different classes of mRNA, thus generating adaptive translatomes from pre-existing mRNA pools. In fact, under hypoxic conditions, the expression of NDRG1 and other hypoxic stress response genes can be regulated not at the transcriptional level, but through the control of translation efficiency by eIF4F^H^. The expression of these hypoxia target proteins, including NDRG1, also increases in the presence of the transcription inhibitor, actinomycin D. This demonstrates that translation efficiency, rather than mRNA expression, may be the primary mechanism of protein level regulation [56].

**Translational control by miRNAs.** NDRG1 protein abundance is also regulated by miRNAs through direct interaction with the 3′ UTR of *NDRG1* mRNA. For example, in a comprehensive analysis of miRNAs modulated during reoxygenation, the authors identified 43 oxygen-sensitive miRNAs in MCF-7 BC cells. Among these oxygen-sensitive miRNAs, miR-769-3p directly inhibits NDRG1 [57]. In CRC, transcriptomic analyses and functional validation have shown that miRNA-483-3p directly targets NDRG1, activating the ERBB3/AKT axis [58]. Additionally, miR-182 promotes prostate cancer cell proliferation and invasion by directly targeting the 3′-UTR of NDRG1 [59].

**Protein degradation**. Several examples of NDRG1 regulation during protein degradation have been reported. In BC cells, lncRNA N-Myc Downstream Regulated Gene 1-Overlapping 1 (NDRG1-OT1) destabilises NDRG1 by promoting ubiquitin-mediated proteolysis [60]. NDRG1 is post-translationally modified by a Small Ubiquitin-like Modifier (SUMO), preferentially by SUMO-2, at Lys14. SUMO-2 modification does not affect the subcellular distribution of NDRG1 but influences its protein stability. NDRG1 SUMOylation functions as a signal for polyubiquitination and degradation [61].

**Figure 3:**
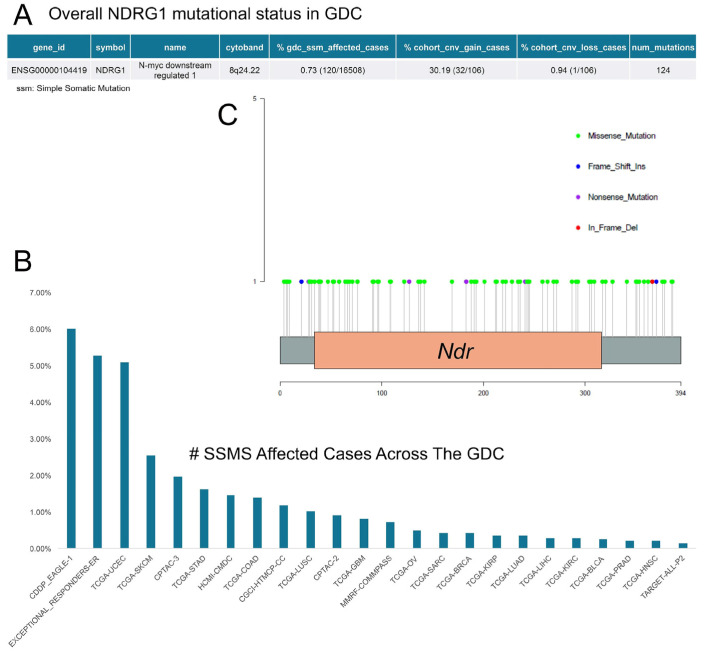
NDRG1 mutational status in GDC projects (**A**) Overall mutation status is reported. (**B**) Alteration rate across the GDC datasets. (**C**) Lolliplot showing the distribution of alterations across protein domains. Each mutation is depicted as a ‘lollipop’, positioned according to its amino acid coordinate and coloured by its predicted functional impact. The height of each lollipop stem represents the frequency of the alterations. Abbreviations: ssm, single-nucleotide mutation; cnv, copy number variation (data were downloaded from the GDC portal and graphically displayed using the ggplot2 R package. Data downloaded on 15 January 2025). CDDP-EAGLE-1, cisplatin (CDDP) response within the Environment And Genetics in Lung cancer Etiology (EAGLE) cohort; EXCEPTIONAL_RESPONDERS, Exceptional Responders Initiative (studies patients with unusual responses to therapies); TCGA-UCEC TCGA, Uterine Corpus Endometrial Carcinoma; TCGA-SKCM, TCGA Skin Cutaneous Melanoma; CPTAC, Clinical Proteomic Tumour Analysis Consortium; TCGA-STAD, TCGA, Stomach Adenocarcinoma; HCMI-CMDC, Human Cancer Models Initiative (HCMI)-Cancer Model Development Center (CMDC); TCGA-COAD, TCGA Colon Adenocarcinoma; CGCI-HTMCP-CC, Cancer Genome Characterization Initiative-HIV+ Tumour Molecular Characterization Project (HTMCP)-Clinical Cohort (CC); TCGA-LUSC, TCGA Lung Squamous Cell Carcinoma; TCGA-CHOL, TCGA Cholangiocarcinoma; TCGA-GBM, TCGA Glioblastoma Multiforme; MMRF-COMMPASS, MMRF-CoMMpass Study (Multiple Myeloma); TCGA-OV, TCGA Ovarian Serous Cystadenocarcinoma; TCGA-SARC, TCGA Sarcoma; TCGA-BRCA, TCGA Breast Invasive Carcinoma; TCGA-KIRP TCGA, Kidney Renal Papillary Cell Carcinoma; TCGA-BLCA TCGA, Bladder Urothelial Carcinoma; TCGA-PRAD TCGA, Prostate Adenocarcinoma; TCGA-HNSC TCGA, Head and Neck Squamous Cell Carcinoma; TARGET-ALL-P2 TARGET, Acute Lymphoblastic Leukaemia (Phase 2).

**Genetic alterations.** The effect of copy number alterations on mRNA and protein abundance has been described. In BC, NDRG1 expression is associated with *NDRG1* gene amplification. The gene is located on chromosome arm 8q, a region frequently gained in primary and metastatic BCs. *NDRG1* is frequently gained (three copies) or amplified (greater than three copies) in primary and metastatic BCs, and this is also associated with increased NDRG1 expression [62]. Data on somatic variations in the Genomic Data Commons (GDC) portal (https://portal.gdc.cancer.gov/, accessed on 20 January 2025) were used to elucidate the mutational landscape of *NDRG1*. The overall incidence of single-nucleotide mutations (SNM) was 0.73% in the repository, with 124 distinct alterations spanning all genes, with missense mutations being the most represented (Figure 3A–C). Copy number variation (CNV) gain and loss were observed in 30.19% and 0.94% of cases, respectively (Figure 3A). The *NDRG1* mutational status was evaluated across the different datasets present in the GDC portal. As reported in Figure 3B, the rate of *NDRG1* mutations was higher in the CDDP_EAGLE-1, EXCEPTIONAL_RESP, and TCGA-UCEC cohorts, including data from the Environment and Genetics in Lung Cancer Etiology study, patients with lasting clinical response, and uterine corpus endometrial carcinoma patients, respectively.

## 3. Functional Roles of NDRG1

### 3.1. NDRG1 Regulation of Cell Differentiation

Studies conducted under different pathophysiological conditions support the hypothesis that NDRG1 expression is involved in cell differentiation. In CRC, the modulation of NDRG1 expression is linked to cellular differentiation processes and the metastatic phenotype. In vitro studies have demonstrated that factors influencing the state of cell differentiation, including ligands of PPARγ, RXR, DNA methylation, and histone acetylation inhibitors, modulate NDRG1 expression. Moreover, NDRG1 overexpression inhibits in vitro invasion through Matrigel and liver metastasis in nude mice [63]. In prostate cancer, NDRG1 expression is inversely correlated with grade and overall survival. Patients with lymph node or bone metastases exhibited significantly reduced NDRG1 expression compared to those with localised disease, suggesting a role for NDRG1 in suppressing metastatic progression. In the normal colon, NDRG1 is expressed in the cytoplasm and basolateral membranes of surface epithelial cells lining the gut lumen. However, mRNA expression is decreased in colon adenomas and adenocarcinomas, suggesting involvement in the differentiation process. This and other studies support the role of NDRG1 as a potential tumour suppressor in human colon and prostate cancer [64,65].

Under physiological conditions, NDRG1 promotes adipogenesis by enhancing the expression of peroxisome proliferator-activated receptor gamma (PPARγ). Triacylglycerol accumulation during adipocyte differentiation appears to be regulated mainly by NDRG1 phosphorylation at position 346. Overexpression of the T346A mutant led to markedly lower triacylglycerol accumulation compared to cells overexpressing wild-type NDRG1 [66].

Upregulation of NDRG1 has also been implicated in epithelial-mesenchymal transition (EMT). However, its functional role in this process is still unclear. While some studies have reported a positive correlation between NDRG1 expression and the transition to a mesenchymal phenotype, others have suggested the opposite. In bladder cancer cell lines in which NDRG1 was overexpressed, the protein expression of the epithelial markers cytokeratin 7 and claudin-1 was markedly decreased, whereas the expression of the mesenchymal markers N-cadherin and β-catenin was upregulated. On the contrary, after NDRG1 knockdown, SNAI2, ZEB2, and TWIST1 expression levels were significantly decreased [67]. In cervical cancer cells, NDRG1 overexpression increases the expression of the mesenchymal marker Vimentin and reduces the expression of E-cadherin [68].

In another study, the blockade of NDRG1 initiates EMT and enhances TGF-β signalling in nasopharyngeal cancer cells. In this cell model, NDRG1 acts as a tumour suppressor by preventing nasopharyngeal tumorigenesis and metastasis by inhibiting Smad2-mediated EMT of nasopharyngeal cells [69]. This potential role has also been confirmed in CRC. In this tumour type, NDRG1 regulates EMT through its interaction with caveolin-1. In detail, NDRG1 reduces caveolin-1 protein expression by promoting its ubiquitylation and subsequent degradation via the proteasome. Moreover, this interaction plays a functional role in mediating the suppressive function of NDRG1 in EMT, migration, and invasion in vitro and metastasis in vivo. In human CRC tissues, low NDRG1 expression is always associated with high caveolin 1 expression, and vice versa [70].

In consideration of its anti-oncogenic role, attention has been focused on identifying small molecules capable of inducing the upregulation of NDRG1, including the chelators desferrioxamine (DFO) and di-2-pyridylketone-4,4-dimethyl-3-thiosemicarbazone (Dp44mT). In HT29 and DU145 cells, chelators inhibit TGFβ-induced EMT via a process consistent with NDRG1 upregulation [71]. Among the mechanisms that explain the functional role of NDRG1, the inhibition of the PI3K/AKT/LYRIC/c-Myc pathway appears to play a central role. Indeed, incubation of DU145 and HT29 cells with tumour necrosis factor alpha (TNFα) was found to induce the expression of LYRIC and EMT, and this effect was abolished in NDRG1 overexpressing cells [72].

### 3.2. NDRG1 Regulation of Vesicular Trafficking

Studies on prostate cancer cells have shown an interaction between NDRG1 and Rab4a, as well as their localisation in recycling/sorting endosomes. In these models, silencing NDRG1 resulted in a decrease in E-cadherin protein levels, suggesting that NDRG1 stabilises E-cadherin via Rab4a [73]. Furthermore, microarray analysis comparing cancer cells overexpressing NDRG1 to those with normal expression levels revealed that NDRG1 is linked to a network of vesicle transport proteins [42]. Functionally, NDRG1 appears to regulate the expression of proteins involved in the endosomal-reticulum axis, thereby reducing secretory or endocytic activity under stress.

### 3.3. NDRG1, ER Stress, and Autophagy

NDRG1 plays a key role in autophagy and ER stress. In pancreatic cancer (PC) cells, different ER stress stimuli (tunicamycin and serum starvation) induce autophagy through a mechanism involving the PERK/eIF2 axis of UPR. PERK/eIF2 is one of the three ER stress signalling pathways (inositol-requiring kinase 1α (IRE1α), activating transcription factor 6 (ATF6), and PERK) that are activated to modulate the ER. NDRG1 suppresses the activation of tPERK/eIF2 and the subsequent autophagy-induced ER stress formation of autophagosomes, increasing cancer cell susceptibility to apoptosis [74].

A subsequent study further demonstrated that NDRG1 may also affect other arms of the ER stress response. NDRG1 increases the expression of three major ER chaperones (binding immunoglobulin protein (BiP), calreticulin, and calnexin), inhibits IRE1α, and increases the cleavage of ATF6 arms of UPR [75].

In agreement with the data obtained in pancreatic cells, in metastatic BC cells treated with metixene, upregulation of total NDRG1 and phospho-NDRG1 induces incomplete autophagy, leading to caspase-mediated apoptosis in both primary and brain-metastatic cells. Upon NDRG1 KO, autophagy was completed, and caspase-mediated apoptosis was not activated [76]. Overall, these studies support the role of NDRG1 as a tumour suppressor. These results, however, have not been confirmed in other tumour models, where the activation state of the UPR is higher in more aggressive cancer cells in vitro and in vivo. In triple-negative Breast Cancer (TNBC), XBP1 promotes cancer development, and in these models, it acts together with HIF as a positive regulator of NDRG1 expression [77]. This suggests that the role of NDRG1 in autophagy and UPR is context-dependent and should be considered in relation to specific cell and tumour types.

### 3.4. NDRG1 Regulation of Senescence/Ageing

In hepatocellular carcinoma (HCC), NDRG1 suppression is correlated with the activation of a senescence-associated signalling pathway. NDRG1 suppression by RNAi induces cellular senescence in HepG2 cells, as demonstrated by the upregulation of p53, p21, and p16 and the decreased phosphorylation of Rb. In human HCC samples, high NDRG1 expression was associated with low p21 and p16 expression. NDRG1 regulates senescence through the GSK-3β–p53/p16 pathway, as cellular senescence induced by NDRG1 suppression is partially prevented by blocking the GSK-3β–p53/p16 pathway in HepG2 cells [78].

The specific role of NDRG1 in ageing has yet to be defined. Ex vivo data obtained from cerebral cortex tissues of subjects with Alzheimer’s disease (AD) support an increase in NDRG1 phosphorylation levels, indicating a possible increase in SGK1 kinase activity. These results support the protective role of the SGK1/NDRG1 pathway in AD [79]. This agrees with experimental observations demonstrating that the activation of the mTORC2 pathway and inhibition of the mTORC1 pathway have therapeutic potential in patients with AD [80].

### 3.5. NDRG1 Regulation of Endothelial Function

NDRG1 regulates endothelial cell (EC) function in response to different stimuli. For instance, flow shear stress is associated with the upregulation of NDRG1 in ECs [81]. Moreover, NDRG1 is significantly upregulated in cytokine-stimulated ECs and in inflammatory aortic tissues from patients with atherosclerotic. Knockdown of NDRG1 inhibits the expression of pro-inflammatory cytokines and prevents the cytokine-induced prothrombotic function in ECs. These effects are likely a consequence of Nur77 upregulation and subsequent inhibition of NF-κB and AP-1 [82].

Watari and coworkers reported a functional link between NDRG1 and angiogenesis. NDRG1 has been shown to regulate angiogenesis through phospholipase C gamma 1 (PLCγ1) signalling in mouse ECs. In VEGF-A-stimulated endothelial cells, NDRG1 forms a complex with PLCγ1 and coordinates its trafficking to the cell membrane, resulting in increased IP3, DAG, and cytoplasmic Ca^2+^ levels and subsequent activation of the ERK pathway. NDRG1 deficiency in ECs selectively interferes with PLCγ1 phosphorylation by VEGF-A, thus preventing ERK activation [83]. This has functional consequences in the pathological context. In high-grade gliomas (HGG), *NDRG1* levels are associated with both microvessel density (MVD) and vasculogenic mimicry (VM). MVD and VM are closely associated with tumour growth, invasion, and metastasis. In fact, Yang H et colleagues showed a significant correlation between VM expression and NDRG1 gene expression and tumour grade, and a probable regulation of tumour MVD by *NDRG1* [84]. These data highlight the pivotal role of NDRG1 in tumour aggressiveness and progression.

### 3.6. NDRG1 Regulation of Myelination

**Figure 4:**
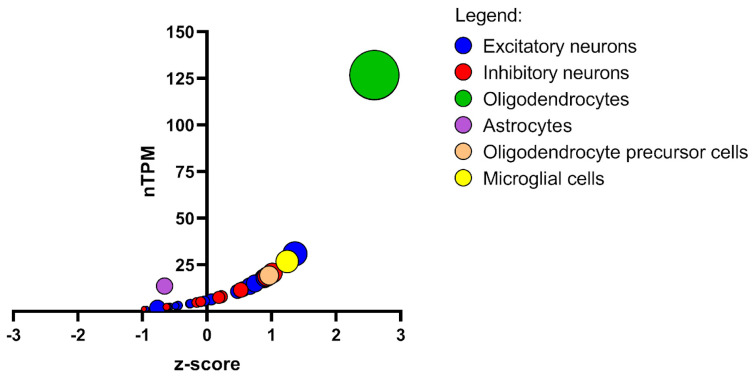
The bubble plot highlights the expression profile of NDRG1 across distinct cell-type clusters derived from RNA-seq analysis. The x-axis represents the z-score, reflecting the normalised expression level of NDRG1 within each cluster. A positive z-score denotes an expression above the mean, whereas a negative z-score indicates a below-mean expression relative to the overall expression distribution across clusters. This standardisation facilitates a direct comparison of the relative expression levels. The y-axis represents the nTPM (normalised transcripts per million), a quantitative measure of the absolute expression level of NDRG1 in each cluster, indicative of transcriptional activity. The bubble size corresponds to the Max-Norm value, representing the fraction of the maximum expression achieved by NDRG1 within each cluster. Larger bubbles highlight clusters where NDRG1 expression approaches its peak, emphasising its relative significance in these cell populations. Data were obtained from the Human Protein Atlas (https://www.proteinatlas.org/, accessed on 20 January 2025) and graphically displayed using GraphPad 9.0 (https://www.graphpad.com/, accessed on 13 January 2025).

Several studies have underscored the role of NDRG1 in regulating myelin sheaths. This conclusion stems from the causative link between NDRG1 and the progression of myelin-related diseases, as well as investigations in mouse models that elucidated its functional role in this context. In the peripheral nervous system, NDRG1 has a specific role in maintaining the myelin layer. Other members of the NDRG family appear to have distinct, non-redundant biological roles. Notably, in animal models with induced NDRG1 deficiency, the levels of other isoforms remain unchanged [85]. Data from the Human Cancer Atlas (https://www.proteinatlas.org/ENSG00000104419-NDRG1/single+cell/brain, accessed on 20 January 2025) further supports the role of NDRG1 in specific cell populations, as illustrated in Figure 4. The bubble plot highlights the differences in NDRG1 expression across cell-type clusters with distinct patterns of transcriptional activity, providing a detailed overview of the expression dynamics of NDRG1 and its potential functional relevance in specific cell populations.

A truncating mutation in NDRG1 is responsible for hereditary motor and sensory neuropathy–Lom (HMSNL), also known as Charcot-Marie-Tooth disease (CMT4D). Patients with the R148X mutation present with peripheral nervous system involvement and central nervous system (CNS) white matter abnormalities [86,87]. Ndrg1^−/−^ mice exhibit a progressive demyelinating disorder of the peripheral nerves, with demyelination at about 5 weeks of age [85].

As a defect in lipid trafficking has been proposed to play a major pathogenic role in HMSL, NDRG1 has been functionally associated with vesicular trafficking, as confirmed by in vitro and in vivo studies. In detail, experimental data correlate the expression of NDRG1 with the trafficking of lipoproteins [88,89]. In epithelial cells, NDRG1 silencing reduces the uptake of low-density lipoprotein (LDL) by reducing LDL receptor (LDLR) abundance at the plasma membrane. Moreover, the specific interaction between NDRG1 and APOA1 and APOA2 was identified using the yeast two-hybrid experimental system and confirmed in mammalian cells. A study conducted in a mouse model of CMT4D with total Ndrg1 deficiency demonstrated that NDRG1 is probably involved in the regulation of ECM remodelling, lipid metabolism and transport, Schwann cell development, myelin proteins, cytoskeleton proteins, inflammation, cell cycle, and signalling [88].

NDRG1 is expressed in the optic nerve and sciatic nerve. NDRG1 immunoreactivity has not been detected in motor and sensory neurons [90]. High expression levels have been observed in Schwann cells (SCs). A dual role has been proposed for NDRG1 in SC: (i) growth arrest and cell differentiation, and (ii) control of lipid biosynthetic pathways essential for glial cell function. In injured sciatic nerve mice, NDRG1 was expressed in the intact mouse sciatic nerve, and double immunofluorescent labelling with the SC marker (S-100) or an axonal marker (NF) showed that NDRG1 was localised in the cytoplasm of SCs, but not in the axons. NDRG1 may be involved in the regeneration of injured nerves as NDRG1 expression is maintained in the early stage of myelin degradation but is markedly reduced at the end stage of myelin degradation [91,92].

During myelination, phospho- and total NDRG1 levels are robustly upregulated. However, phosphorylation is compartment-specific. Total and phosphorylated NDRG1 isoforms are enriched in the Cajal bands and clefts, whereas p-NDRG1 is enriched in the abaxon of myelinating SC. This membrane region mediates interactions with laminin in the basal lamina, notably integrins, including a6b1 and a6b4. Consistent with the proposed role of NDRG1 and a6b4 during myelination, a6b4 integrin regulates NDRG1 phosphorylation through a signalling pathway that involves PI3-K/AKT/SGK1 [93].

## 4. Conclusions

In recent years, there has been growing scientific interest in NDRG1, given its role in various physiological and pathological contexts. NDRG1 can regulate differentiation, cell cycle, stress response, angiogenesis, vesicular trafficking, and myelination. In pathological contexts, NDRG1 is considered an independent prognostic marker in different tumour types, and *NDRG1* gene mutations have been identified as causal factors of genetic diseases. Its expression and activity are tightly regulated through the control of all stages of gene expression and protein turnover and through changes in its functional localisation within the cell. Moreover, the mechanisms that regulate NDRG1 function via post-translational modifications respond to multiple signal transduction pathways and external stimuli. An important functional response to post-translational modifications of NDRG1 is a change in its intracellular localisation, including the cytoplasm, membrane, mitochondria, or nucleus. As described above, one of the main methods by which cells control NDRG1 distribution is through phosphorylation. This has a consequent effect on the functional role of NDRG1, as certain post-translational modifications appear to have greater effects on the regulation of specific biological processes. For example, phosphorylation of NDRG1 at Ser336 is associated with mitochondrial localisation and regulation of mitochondrial fission and respiration [55], whereas phosphorylation at Thr346 promotes membrane localisation and may assist in the correct distribution of enzymes or receptors [94].

An alternative mechanism that can drive protein compartmentalisation is the generation of alternative isoforms with different localisation signals. For example, NDRG1 has an N-terminal site that guides nuclear localisation. The truncated form of NDRG1 has a predominantly cytoplasmic localisation [6] and reduced protein stability compared to mRNA [95]. In contrast, full-length NDRG1 accounts for the tumour-promoting role of this protein [95].

Despite evidence of its nuclear localisation, the full range of processes regulated after nuclear translocation is difficult to elucidate. However, some biological functions of NDRG1 appear to be strictly associated with nuclear localisation. For instance, the ability to regulate cell motility is dependent on GSK-dependent phosphorylation of the 3R domain, with consequent nuclear translocation [47]. It is possible that NDRG1 acts as a chaperone for the nuclear translocation of specific transcription factors, and several lines of evidence support this notion [96]. For instance, the nuclear localisation of TAF15, a component of RNA polymerase II, decreases after NDRG1 knockdown in human pulmonary artery endothelial cells [97]. Recently, our mass spectrometry (MS/MS) experiments and subsequent gene ontology enrichment analysis of BC NDRG1-Empty and NDRG1-CRISPR cells revealed a reduction in proteins associated with “Nucleocytoplasmic transport” after NDRG1 knockdown, supporting the potential role of NDRG1 as a chaperone [98].

If we consider the cellular mechanisms that govern the expression of NDRG1, most stimuli described above induce protein overexpression predominantly via transcription. Therefore, it can be assumed that protein levels can be predicted by studying *NDRG1* mRNA levels, but with some exceptions. Examples of the dynamic range of protein abundance being regulated via post-transcriptional mechanisms have been described (i.e., during hypoxia). As reported, NDRG1 protein tends to be very stable compared to mRNA in response to perturbation. This difference in mRNA-protein abundance may reflect a specific biological role [99].

Regarding the mutational status of the gene and its effect on expression levels and phenotype, clear literature data exist for CMT. In cancer, the probability of *NDRG1* mutations occurring in tumours is low (Figure 3). The majority of these mutations are missense mutations, and it is unclear whether mutations in *NDRG1* exert a specific functional effect. On the contrary, CNVs mechanisms appear to be more involved in gene amplification processes (Figure 3A). For instance, the amplification of the chromosomal fragment 8q24.2 is associated with amplifications of *MYC* and *NDRG1* located on this fragment and is significantly correlated with homologous recombination deficiency (HRD) across tumour types [100].

Various studies have identified NDRG1 as a clinical indicator of prognosis or recurrence following chemotherapy. Considering the regulatory mechanisms outlined earlier, the significance of RNA–protein correlation may be pertinent in defining the potential clinical value of NDRG1. Given that NDRG1 phosphorylation can reflect its localisation and function, protein levels appear to be more consistent. In a research conducted by López-Tejada and colleagues, both total NDRG1 and p-NDRG1 (Thr346) positivity and subcellular localisation were crucial for the survival of patients with triple-negative breast cancer [52]. In non-small cell lung cancer, elevated levels of p-NDRG1 (Thr346) were associated with decreased overall survival [101]. 

Simultaneously, a retrospective RNA-seq data analysis revealed that NDRG1 mRNA is an independent risk factor for overall survival (OS) in patients with glioma [84]. Overall, the use of mRNA or proteins in the clinical management of cancer has not yet been fully evaluated. Due to the heterogeneity in NDRG1 expression at the single-cell level (Figure 4), the adoption of single-cell RNA-seq may introduce an additional layer of specificity, offering further insight into NDRG1 expression at the single-cell resolution.

The evidence presented above indicates that NDRG1 serves as a tissue biomarker for cancer management. Additionally, data from the Human Protein Atlas demonstrate that this protein is present in the bloodstream, with its concentration significantly increasing in cases of liver diseases, including hepatocarcinoma. This establishes a basis for future studies to ascertain its clinical potential as a tumour biomarker derived from blood. Furthermore, given its vesicular localisation, it remains to be clarified whether NDRG1 can be transported in the bloodstream via extracellular vesicles in future investigations.
